# Prediction Algorithm of Uncertain Fund Demand for Regional Economics Using GM Model and Few-Shot Learning

**DOI:** 10.1155/2022/2307149

**Published:** 2022-07-01

**Authors:** Baoqian Wang

**Affiliations:** School of Economics and Finance, Zhanjiang University of Science and Technology, Zhanjiang 524094, China

## Abstract

The forecast of capital demand has the characteristics of uncertainty. There are known and unknown information about the capital demand for regional economic development. In fact, there are also some in between, that is, uncertain. Consumption is the ultimate goal of production and a key link in realizing a virtuous circle of economic development. This paper uses the GM (1, 1) model to compare the predicted value of the test area with the actual value in 5 years, and the loudness is as high as 90%. Under the guidance of the profit model, the regional economic capital demand has a decisive influence on the regional economic development. The predictive analysis model of capital needs is conducive to fully mobilizing the impact of infrastructure construction of all parties and is an important factor affecting economic development. The mathematical model proposed in this paper is helpful for deepening the research on the management of regional economic development and enriching the theoretical system of regional economic development.

## 1. Introduction

At present, grey system modeling methods are mainly used for grey prediction and decision-making, and have achieved good results. The construction of grey model, through the action of grey generation or sequence operator, weakens randomness, taps potential laws, and realizes a new leap in establishing continuous dynamic differential equations using discrete data sequences through the exchange of grey difference equations and grey differential equations. The grey prediction of the sequence formed by the system behavior characteristic index value is called sequence prediction [1, 2]. In the GM (1, 1) model, GM (1, 1) represents a first-order, one-variable differential equation prediction model, which is a first-order single-sequence linear dynamic model, which is mainly used for time series prediction. For the sequence of system behavior characteristic indicators, through different combinations and trade-offs, multiple derived sequences are formed, and a prediction model is established for it.

It is difficult to distinguish the quantitative relationship between factors and the primary and the secondary relationship between systems among many factors in the objective world due to lack of data, incomplete information, and uncertainty. The goal of grey correlation analysis is to figure out how closely factors in a system are related. The abovementioned freight volume and cargo turnover can both represent capital demand, but which one is more representative? A type of social and economic activity that leads to demand is capital demand. The correlation analysis with the two indicators, expressed in terms of income elasticity value, can determine which indicator is more representative [3, 4]. The main issues arise during the transition from one mode of regional economic development to another. These issues are most visible in the following areas: regional energy consumption is excessive, and the growth mode is extensive, which makes it difficult to adjust and optimize the industrial structure; in the process of rapid regional economic growth, environmental protection issues have not been prioritized, resulting in serious pollution; for a long time, the region's primary sources of income were investment and export. Consumption has been insufficient for a long time, and the development of investment and consumption is uncoordinated; the industrial structure is unreasonable, traditional industries remain dominant, the proportion of high-tech industries is low, there are issues of weak agricultural foundations, low industrial quality, and lagging development of service industries: urban and rural; the industrial structure is unreasonable, traditional industries remain dominant, the proportion of high-tech industries is low; there are problems of weak agricultural foundations, low industrial quality, and lagging development of unbalanced regional development, widening income gaps between residents; low investment in scientific and technological talents, education, and research and development, affecting labour quality improvement; insufficient independent innovation ability, serious lack of independent brands; overseas enterprises' investment difficulties, and so on [5].

The innovation of this paper is to combine the grey system theory method with the classical consumption demand theory and econometric method, conduct a comprehensive analysis of regional consumption demand and regional economic development capital demand, combine the income elasticity value and forecast income elasticity value, and make a comparison; it is concluded that the degree of acquaintance between the predicted value and the actual value is as high as 90%. The structure of this paper is as follows.  Section 1 studies and summarizes the related research on regional economic development theory and grey system model.  Section 2 briefly introduces the concept of grey system model and establishes a mathematical model based on grey system model.  Section 3 puts forward the relationship between regional residents' income and regional economic development, introduces a certain region's consumption expenditure as a sample, and conducts model statistics.  Section 4 predicts and compares sample values based on GM (1, 1) model.  Section 5 summarizes the full text.

## 2. Related Work

With the development of human society, especially the development of industrial revolution and information technology, human's understanding of the connotation of infrastructure has experienced a process of continuous development of levels and scope. In the mid-19th century, Hong et al. put forward the idea of social indirect capital on the basis of summarizing earlier economists' arguments, pointing out that social indirect capital includes all those foundations such as electricity, transportation, or communication industry [6]. Nowak et al. based on uncertainty and rational expectations, put forward the theory of excessive sensitivity, and the theoretical framework of consumption under uncertainty has been initially established [7]. Liu et al. introduced uncertainty into consumption theory for the first time and proposed a random walk model to analyze the influence of uncertainty factors on consumption [8]. Zu et al. put forward a hypothesis theory, which holds that there are two different types of consumers in the economy who choose consumption in each period according to the life cycle hypothesis and decide consumption behavior according to current income [9]. Man and Chen simulated and verified the buffer stock model through the backward method and found that there is a very significant linear relationship between income uncertainty, persistent income, and wealth goals. Consumption structure is also an important aspect that reflects the level of residents' consumption demand [10]. René et al. believe that infrastructure includes two categories: one is core infrastructure, mainly referring to transportation and electricity, whose role is to increase the productivity of physical capital and land, and the other is human infrastructure, including healthcare and education [11]. Ma et al. put forward the idea of social indirect capital on the basis of summarizing the early economists' discussions, pointing out that social indirect capital includes all those basic industries such as electricity, transportation, or communication [12].

It is further pointed out that social indirect capital is divided into broad and narrow senses, and it is believed that in its broad sense, it includes everything from law, order and education, public health to transportation and communication, power, water supply, and agricultural indirect capital such as irrigation and drainage systems. Public service [13]: regarding the development scale of infrastructure, it mainly focuses on the relationship between total public capital expenditure and total output, the relationship between physical stock of infrastructure and total output, infrastructure, and sectoral output, and the difference between infrastructure and regional economic growth. Research: the general conclusion is that there is an obvious elastic coefficient between the total expenditure of infrastructure and the total output [14]. The “priority development theory” advocated by Chen et al. believes that in order to rapidly change the face of economic backwardness, a region must concentrate its energy, invest a large amount of money at one time, and give priority to the development of infrastructure [15]. Zeng and Luo used ELES and AIDS models to compare and analyze the consumption demand of regional residents [16]. Ho et al. studied the evolution mechanism, characteristics, and laws of regional residents' consumption behavior from multiple perspectives, and made an empirical analysis on the influencing factors of regional residents' consumption behavior by combining the western neoclassical consumption theory with the actual situation in China [17] ]. Liu et al. proposed an extended linear expenditure system model that further considers income distribution, and preliminarily constructed a more explanatory rural residents' consumption demand function according to the actual situation of the region. The function theory research has injected new blood [18].

The research results of the above scholars have important reference and reference significance for promoting the theory of capital demand for regional economic development, based on the above literature. However, most consumer demand theory research uses more mature economic theories of the grey system model to conduct exploratory and confirmatory analysis of the actual situation in the region; however, due to data availability and authenticity limitations and some scholars' lack of data processing methods, the results are frequently unsatisfactory, resulting in weak convincing. Furthermore, related researchers share a common research perspective on regional economic consumption demand. Majority of academic research focuses on the structure of economic development capital demand, but there is a lack of in-depth and detailed research on consumption rate and the factors that influence consumption demand. At the same time, some scholars have glaring flaws in their sample data selection or analysis methods.

## 3. Concept and Model Establishment of Grey System

The data model established by using grey theory is not the original data sequence, but the data model generated by a series of processing. The GM (*n*, *m*) model is the basic model of the grey forecasting method, where *n* is the order of the differential equation, and *m* is the number of variables. The GM (1, 1) model is a grey model with a differential equation order of 1 and a variable unit digit of 1. It is the simplest grey prediction model.

The modeling steps of the GM (1, 1) prediction model are as follows:

Suppose the original data sequence is (1):

(1) $$\begin{array}{c} X^{\left(0\right)}=\left(x^{\left(0\right)}\left(1\right),x^{\left(0\right)}\left(2\right),\ldots ,x^{\left(0\right)}\left(n\right)\right). \end{array}$$

Due to the low regularity and large fluctuation of the original data, it will adversely affect the prediction results [19, 20]. Therefore, in the grey prediction, the data is usually processed by the cumulative generation, and the cumulative generation sequence is (2):

(2) $$\begin{array}{c} X^{\left(1\right)}=\left(x^{\left(1\right)}\left(1\right),x^{\left(1\right)}\left(2\right),\ldots ,x^{\left(1\right)}\left(n\right)\right), \end{array}$$

Where, *X*^(1)^(*k*)=∑_*i*=1_^*k*^*x*^(0)^(*i*), *k*=1,2,…, *n* is the number of original data.

Define the data sequence on the basis of the data sequence generated after the accumulation *Z*^(1)^ as *X*^(1)^. The next-to-neighbor mean generation sequence is

(3) $$\begin{array}{c} Z^{\left(1\right)}=\left(z^{\left(1\right)}\left(2\right),z^{\left(1\right)}\left(3\right),\ldots ,z^{\left(1\right)}\left(n\right)\right), \end{array}$$

where *z*^(1)^=1/2(*x*^(1)^(*k*)+*x*^(1)^(*k* − 1)), *k*=2,3,…, *n* is the number of original data.

Then, define the basic form of GM (1, 1) as (4):

(4) $$\begin{array}{c} x^{\left(0\right)}\left(k\right)+az^{\left(1\right)}\left(k\right)=b. \end{array}$$

The parameters in formula (4) are:*a* represents the coefficient of development, the parameter *b* represents the grey action.

Use the least squares estimation method to find (5)

(5) $$\begin{array}{c} {\left[a,b\right]}^{T}={\left(B^{T}B\right)}^{-1}B^{T}Y. \end{array}$$

Perform whitening (6):

(6) $$\begin{array}{c} \frac{dx^{\left(1\right)}}{dt}+ax^{\left(1\right)}=b. \end{array}$$

Get the time response sequence (7):

(7) $$\begin{array}{c} \hat{x}\left(k+1\right)=\left(x^{0}\left(1\right)-\frac{b}{a}\right)e^{-ak}+\frac{b}{a},k=1,2\ldots . \end{array}$$

Although the GM (1, 1) model has made great improvements in prediction accuracy and data requirements than other prediction models, it is easy to cause errors when the original data rules are weak and there are abnormal or missing data. Therefore, this paper establishes an improved grey forecasting model.

Assuming the regional economic development fund demand as a system factor, the data sequence of relevant factors is (8):

(8) $$\begin{array}{c} X_{i}=\left(x_{i}\left(1\right),x_{i}\left(2\right),\ldots ,x_{i}\left(n\right)\right)\;,i=1,2,\ldots . \end{array}$$

Finally, according to the system factors, the evaluation value of the correlation coefficient is obtained, that is, the required correlation degree (9):

(9) $$\begin{array}{c} r_{0i}=\frac{1}{n}\sum_{k=1}^{n}r_{0i}\left(k\right),\;i=1,2,3,\ldots . \end{array}$$

The value of *r*_0*i*_ is between 0 and 1, and the larger the value, the higher the correlation between the modification factor and the feature sequence.

## 4. Construction of Forecasting System of Fund Demand for Regional Economic Development

### 4.1. Analysis on the Composition of Investment and Financing Mechanism of Regional Economic Infrastructure

The essence of the investment and financing mechanism for regional economic infrastructure projects is to promote competition through the reform of the investment and financing system, and to realize that the market-oriented investment and financing mechanism plays a fundamental role in the allocation of various resources for regional economic infrastructure projects [21].

**Figure 1:**
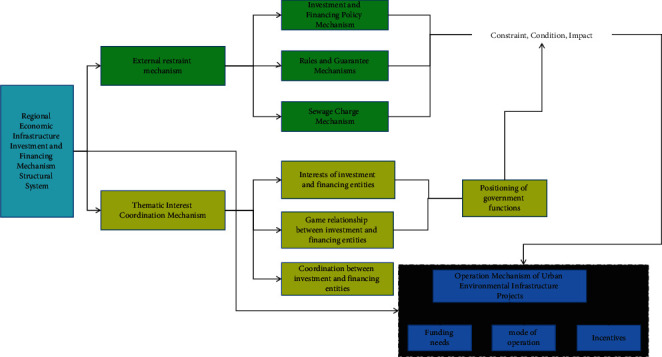
The structural system of regional economic infrastructure investment and financing mechanism.

This paper believes that the construction of a market-oriented investment and financing mechanism system for regional economic infrastructure projects should include three mechanisms: investment and financing external restraint mechanism, investment and financing subject interest coordination mechanism, and investment and financing operation mechanism, based on the logical structure of investment and financing operation of regional economic infrastructure projects. The external investment and financing mechanism is the external environment constraint mechanism for the operation of regional economic infrastructure investment and financing projects, and it is the existence condition and foundation of the regional economic infrastructure investment and financing mechanism. Policy mechanisms, institutional guarantee mechanisms, and payment mechanisms are all included. The investment and financing subjects' interest coordination mechanism is a game and is a coordination mechanism for the behavioral constraints of investment and financing subjects of regional economic infrastructure projects, as well as their own interests. In the process of investing in and financing regional economic infrastructure projects, the investment and financing operation mechanism is the financing demand, investment, and financing channel and method selection, and operation procedure mechanism. External mechanisms for investment in and financing of regional economic infrastructure projects, as well as a mechanism to coordinate the interests of investment and financing entities and an investment and financing operation mechanism, are required [22]. The investment and financing mechanism is shown in Figure 1.

### 4.2. The Relationship between Economic Development and Consumer Demand

The demand for funds for economic development is reflected not only in terms of quantity but also in terms of consumption structure. The absolute quantity of various consumer goods reflected in a certain form of measurement (physical form or value form, this paper uses value form) is referred to as consumption quantity; the proportion of various consumer goods in total consumption is referred to as consumption structure. The entire process of changes in the level of consumption demand is accompanied by changes in consumption quantity and structure. They are two aspects of consumption demand that are complementary to each other. The upgrading of the consumption structure often leads to the increase in consumption quantity, and the increase in consumption quantity in turn promotes the upgrading of the consumption structure, so that the level of consumption demand develops in a fast and positive direction. The general rule governing the evolution of residents' consumption structures is that when income levels are low, people's consumption expenditures go toward food, clothing, and other necessities. The proportion of consumption expenditures dedicated to development and enjoyment will skyrocket [23].

**Figure 2:**
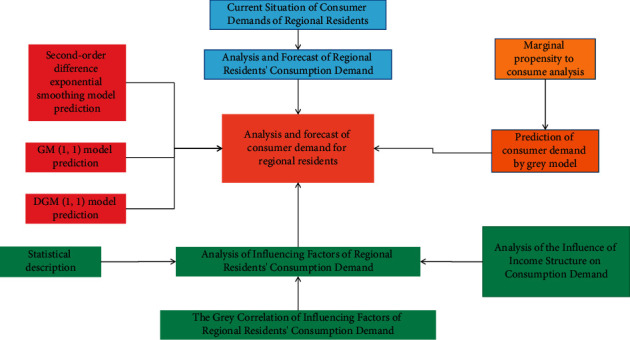
Investment and financing mechanism model of regional consumption demand and economic development.

General principles of economics state that economic activity is the most efficient only when the private rate of return on an investment is close to or equal to the social rate of return. Therefore, the efficient economic development of regional consumer demand projects should also follow such a rule and strive to make various investment entities obtain their reasonable expected returns when investing in regional consumer demand. In the practice of economic development of regional consumer demand projects, the source of funds can include both domestic capital and international capital. The main bodies of domestic capital include governments, enterprises, financial institutions, residents, and non-profit institutions, while international capital includes governments, financial institutions, enterprises, and individuals of various countries. As the main body of economic development, the government focuses more on obtaining social benefits from the construction of regional consumer demand, while other economic development bodies are relatively more willing to obtain economic benefits. The combination of the two constitutes the total expected return of investment. When the actual income from the completion of the regional consumer demand project is close to the total expected income of each investment entity, it is a feasible investment behavior in theory. Financing portfolio structure, resulting in various financing models, is shown in Figure 2.

As shown in Figure 2, the size of the economic contribution (benefit) of the consumer demand infrastructure project is reflected in the degree of satisfaction that its output products bring to consumers. The monetary performance of this degree of satisfaction is the consumer, that is, including all stakeholders. Willingness to pay: under the condition of market economy, the willingness to pay price is the shadow price of the product, and the willingness to pay of consumers for the output of the project is the contribution of the project to the economy. Consumer willingness to pay can be further divided into two parts: actual consumer payment and consumer surplus. Consumer surplus is the balance of the currency that consumers are willing to pay more than the currency actually paid when purchasing a certain commodity.

Consumption demand refers to the various types of consumption materials consumed by residents in the process of consumption under certain social and economic conditions, which can be divided into two types: physical form and value form. Among them, the physical form is the most basic and primitive form of various types of consumption materials consumed in the consumption process represented by the physical quantity; and the value form is represented by currency in the consumption process and the types of consumption data, which are embodied in various living consumption expenditures in real economic life. By analyzing the value of various types of consumption data consumed by regional residents, the development level and changing trend of regional residents' consumption demand at different time periods are revealed. The consumption demand of various types of consumer goods of regional residents analyzed in this paper is expressed in the form of value.

### 4.3. Statistical Data Selection of Regional Residents' Consumption Demand

Since it is not easy to distinguish developmental and enjoyment expenditures, this article will facilitate the forecasting and calculation of regional economic development capital needs in the future. Referring to the consumption expenditure statistics of a certain place, the living consumption expenditures of residents in the modified area are divided into food, clothing, housing, household equipment, transportation and communication, and culture, education and entertainment, healthcare, and other goods and services. The specific statistics are shown in Tables 1 and 2.

## 5. Consumption Demand Forecast Based on GM (1, 1)

### 5.1. Model Parameter Estimates and Errors

**Figure 3:**
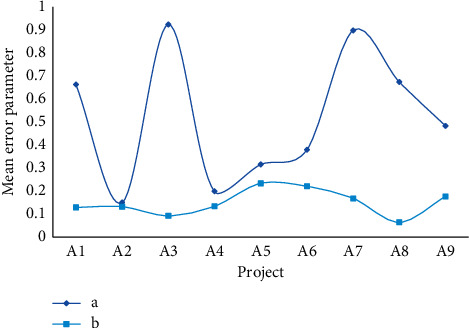
Error parameters of parameters *a* and *b*.

From Table 1, it can be seen that the housing expenditure data in the eighth year is abnormal, which is not increased but decreased compared with the previous data. This abnormal data should be excluded when using the GM (1, 1) model for simulation. Taking the horizontal data in Table 1 as the original time series data, a GM (1, 1) model is established, respectively, and the average relative error of parameters *a* and *b* can be obtained as shown in Table 3 and Figure 3.

**Figure 4:**
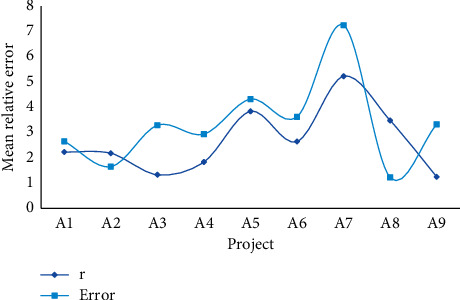
Average error parameters.

It can be seen from Figure 3 that the absolute value of the development coefficient a is less than 0.3, so GM (1, 1) can be used for medium- and long-term forecasting. The correlation degree and error are shown in Table 4 and Figure 4.

It can be seen from Figure 4 that the average relative error of other items except traffic communication is less than 5%, so the fitting accuracy is high. The average relative error of traffic communication is larger, reaching 7.245%, but it is within the acceptable range.

### 5.2. Model Based Parameter Estimation

**Figure 5:**
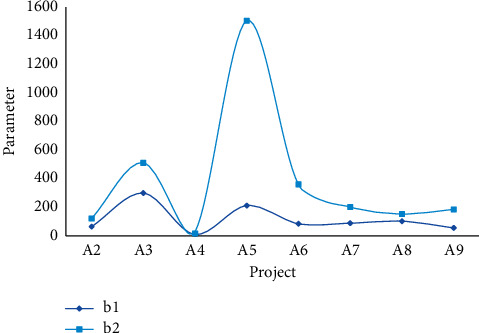
Comparison of parameter *b* samples and predictions.

**Figure 6:**
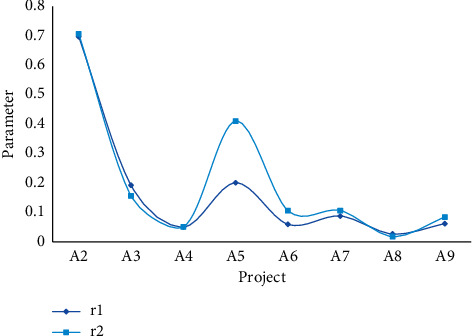
Comparison of parameter *r* samples and predictions.

The data required by the grey system model are the per capita net income of regional households, the total expenditure of living consumption, and the expenditure of its various components. From the data in Tables 1 and 3, the parameters *b* in Figure 3 are estimated by the least squares method in the mathematical model established above. The results are shown in Table 5, Figures 5 and 6.

It can be seen from the table that with the continuous improvement of income level, the consumption desire of regional residents has increased significantly. The marginal propensity to consume of regional residents' total living consumption expenditure increased from 0.6970 in the sample period to 0.7068 in the forecast period. This shows that regional residents will use 70.68% of the new net income for consumption; that is to say, for every RMB 1 increase in disposable income, regional residents will spend RMB 0.7068 for consumption. And the new net income is mainly used for food and housing consumption, which will help to improve the diet and living standards of regional households. It can also be seen from the table that the marginal propensity to consume of regional residents has grown the fastest, from 0.2007 in the sample period to 0.4103 in the forecast period, more than doubling. This shows that with the improvement of living standards, regional residents have a strong demand for the improvement of living conditions after the basic satisfaction of food and clothing. Followed by household equipment supplies and services, which increased from 0.0594 to 0.1059, an increase of 78.28%. This shows that the residents of the region are paying more attention to the improvement of basic living needs such as clothing, food, and housing, and at the same time, they are also pursuing more development-oriented and enjoyment-oriented consumption. Because durable household equipment such as washing machines, refrigerators, air conditioners, shower water heaters, mobile phones and color TVs, video cameras, home computers, and medium- and high-end musical instruments not only bring more enjoyment in life to the residents of the vast area, but also its own development has a great role in promoting. Therefore, the government should correctly guide the consumption of durable consumer goods by regional residents, continue to strengthen policies to strengthen and benefit farmers such as “home appliances to the countryside,” and strive to improve the overall quality of regional residents while promoting domestic demand. In addition, the marginal propensity to consume for transportation, communication and healthcare also increased by varying degrees. Transportation and communication increased from 0.0875 to 0.1059, an increase of 21.03%; healthcare increased from 0.0616 to 0.0836, an increase of 35.71%. This shows that the regional residents' consumption demand for these two items has also increased. More travel, exchanges, and attention to physical health will enable them to develop their spirit and body well, and contribute to the construction of a new socialist region and lay the foundation for cultivating more new types of residents. However, the marginal propensity to consume of food and cultural, educational, and recreational goods and services has decreased by varying degrees. Food decreased from 0.1924 to 0.1563, a decrease of 18.76%; cultural, educational, and recreational goods and services decreased from 0.0268 to 0.0181, a decrease of 32.46%. The downward trend in the marginal propensity to consume food items is expected. Because, at this stage, the food needs of regional residents have been basically met and will be gradually increased. Therefore, with the basic satisfaction of regional residents' food consumption needs, their desire for food consumption needs will decrease. This is consistent with the Law of Diminishing Marginal Utility stage 3 and is basically consistent with the current situation in the region. The marginal propensity to consume of cultural, educational, and recreational goods services is the lowest in both the sample period and the forecast period, and continues to decline. It can be seen that the current consumption of cultural, educational, and recreational articles and services by regional residents is still at a very low level and has a downward trend, which may be closely related to the consumption habits of regional residents who focus on food consumption and less on spiritual consumption.

### 5.3. Regional Economic Development and Income Elasticity Analysis

When the income changes by 1% and the price remains unchanged, the percentage of change in consumption demand is an important indicator of the capital demand for regional economic development. *w*_*k*_ is the income elasticity of item k consumer goods demand, as shown in (10) .

(10) $$\begin{array}{c} w_{k}=\frac{dV_{k}}{dY}\frac{Y}{V_{k}}=\frac{\beta_{k}Y}{V_{k}}. \end{array}$$

**Figure 7:**
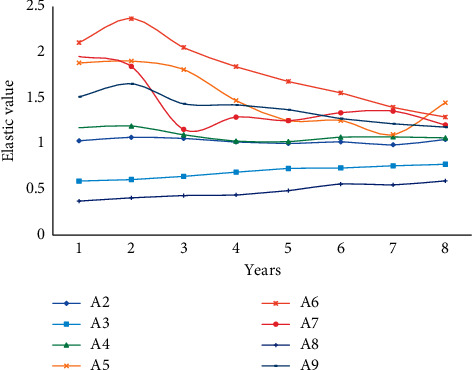
Income elasticity of regional residents during the sample period.

**Figure 8:**
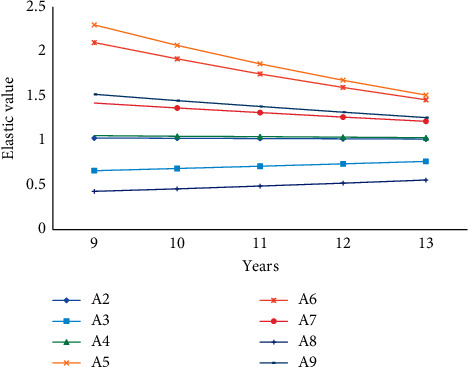
Income elasticity of regional residents during the forecast period.

From the data in the table below and according to formula (10), the income elasticity of various consumption demands of regional residents in each year of the sample period and the forecast period can be calculated as shown in Table 6, Figures 7 and 8.

The income elasticity of regional residents' total consumption demand during the sample period and the forecast period is greater than 1, with the exception of the seventh year, indicating that regional residents' overall consumption demand is growing faster than their income. The income elasticity of consumer demand for clothing, residence, household equipment supplies and services, transportation and communication, and medical care is significantly greater than 1, indicating that regional residents' consumption demand for these types of goods and services is significantly higher than their income growth rate; the two items of household equipment, supplies, and services are significantly higher than the other items, indicating that regional residents' consumption demand for these types of goods and services is significantly higher than their income growth rate; and the two items of household equipment, supplies, and services are significantly higher. As a result, first, in the coming years, such consumer goods will become a powerful lever for leveraging consumer demand, contributing more to economic development. Second, the income elasticity of consumer demand for food and cultural, educational, and recreational goods and services are all less than 1, indicating that these two consumption demands are growing at a slower rate than residents' income; in particular, the income elasticity of consumer demand for cultural, educational, and recreational goods and services is much lower than 1. It demonstrates that regional residents' demand for such consumption is currently growing at a much slower rate than their income.

**Figure 9:**
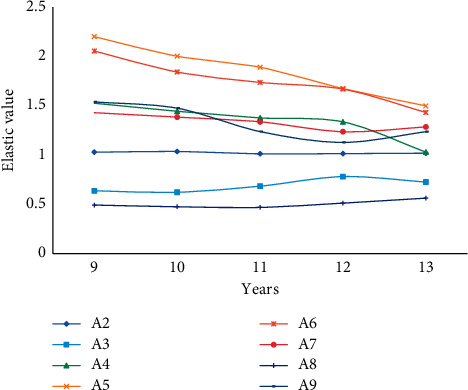
Income elasticity of residents in the sample area from 9 to 13 years.

Based on this, we select the statistics of residents' consumption income and expenditure in the modified region from 9 to 13 years and compare it with the predicted elasticity value as shown in Figure 9.

No matter in the sample period or the forecast period, the income elasticity of consumer demand for each item whose income elasticity of consumption demand is greater than 1 shows a downward trend; at the same time, the income elasticity of consumption demand is less than 1 for food, cultural, educational, and recreational goods and services. However, the income elasticity of consumer demand shows a clear upward trend; thus, the total income elasticity of consumer demand tends to converge toward 1. The similarity between the predicted income elasticity value and the actual elastic growth value is 90%. With the development of the regional economy and the improvement of the living standards of the regional residents, the growth rate of capital demand will keep pace with the income growth.

This paper uses the grey relative correlation analysis method to analyze several main influencing factors that affect regional residents' economic development demand, based on the grey system model theory and the more representative economic development demand theory. The actual elasticity growth value is 90% similar to the predicted income elasticity value. Because it is difficult to find reasonable quantitative indicators for individual factors and data for some indicators is scarce, this paper analyses influencing factors such as regional residents' per capita net income, regional residents' economic development price index, urbanization rate, and per capita GDP. The findings show that each factor has a different impact on regional residents' economic development spending. Because per capita net income is the most important factor influencing economic development, this paper also examines the income structure of regional residents and the impact of various sources of income on economic development requirements. The findings show that net income from family businesses has the greatest impact on regional residents' economic development needs, followed by wage income, and property and transfer income. When regional residents' overall income growth is slow or difficult, improving the income structure can help to promote economic development.

## 6. Conclusions

Since some statistical data are difficult to obtain, this paper uses time series data instead of panel data when selecting sample points. Therefore, this study cannot reflect the economic development needs and development trends of regional residents of different income levels. In further research, we can consider selecting relevant panel data of regional residents at different income levels. For example, regional residents can be divided into low-income groups, middle-low-income groups, middle-income groups, middle-high-income groups, and high-income groups according to their income levels. Each of the five income levels are analyzed, and the economic development needs of different income levels of regional residents are specifically analyzed, and efforts are made to study the needs and future development trends of regional residents at different levels for different types of economic development in more detail.

## Figures and Tables

**Table 1:** Average per capita net income and living consumption expenditure of residents in a certain region in 8 years (unit: yuan).

Project/year	1	2	3	4	5	6	7	8
Net income per capita (*A*1)	2236	2553	2871	3261	3852	4454	4807	5524
Living consumption expenditure (*A*2)	1509	1664	1892	2229	2676	3044	3388	3682
Food (*A*3)	727	808	859	911	1017	1166	1220	1371
Clothing (*A*4)	96	108	132	160	190	210	226	262
Residence (*A*5)	238	269	318	444	616	713	876	765
Household equipment supplies (*A*6)	63	64	83	105	136	170	204	254
Communications (*A*7)	100	121	160	221	269	291	310	401
Culture, education and entertainment (*A*8)	161	168	178	199	212	214	234	250
Healthcare (*A*9)	91	95	123	141	173	215	243	288
Other goods and services (*A*10)	33	31	39	49	62	66	76	90

**Table 2:** Composition of per capita living consumption expenditure of residents in a certain region in 8 years (unit: %).

Project/year	1	2	3	4	5	6	7	8
Living consumption expenditure (*A*2)	100	100	100	100	100	100	100	100
Food (*A*3)	48.18	48.56	45.4	40.87	38	38.3	36.01	37.24
Clothing (*A*4)	6.36	6.49	6.98	7.18	7.1	6.9	6.67	7.12
Residence (*A*5)	15.77	16.17	16.81	19.92	23.02	23.42	25.86	20.78
Household equipment supplies (*A*6)	4.18	3.85	4.39	4.71	5.08	5.58	6.02	6.9
Communications (*A*7)	6.63	7.27	8.46	9.91	10.05	9.56	9.15	10.89
Culture, education and entertainment (*A*8)	10.67	10.1	9.41	8.93	7.92	7.03	6.91	6.79
Healthcare (*A*9)	6.03	5.71	6.5	6.33	6.46	7.06	7.17	7.82
Other goods and services (*A*10)	2.19	1.86	2.06	2.15	2.35	2.14	2.21	2.47

**Table 3:** Average error parameters of parameters *a* and *b*.

Project	*A*	*B*
Net income per capita (*A*1)	0.6623	0.1284
Living consumption expenditure (*A*2)	0.1497	0.1312
Food (*A*3)	0.9232	0.0919
Clothing (*A*4)	0.1986	0.1336
Residence (*A*5)	0.3151	0.2328
Household equipment supplies (*A*6)	0.3792	0.2201
Communications (*A*7)	0.8973	0.1674
Culture, education and entertainment (*A*8)	0.6736	0.0639
Healthcare (*A*9)	0.4828	0.1759

**Table 4:** Average relative error (unit: %).

Project	*r*	Error
Net income per capita (*A*1)	2.235	2.657
Living consumption expenditure (*A*2)	2.184	1.654
Food (*A*3)	1.336	3.291
Clothing (*A*4)	1.842	2.941
Residence (*A*5)	3.842	4.325
Household equipment supplies (*A*6)	2.648	3.626
Communications (*A*7)	5.231	7.245
Culture, education and entertainment (*A*8)	3.482	1.228
Healthcare (*A*9)	1.254	3.327

**Table 5:** Estimated values of the parameters of the living consumption expenditure of regional residents.

Project	1–8 years sample period		9–13 forecast period	
Net income per capita (*A*1)	*b*1	*r*	*b*2	*r*
Living consumption expenditure (*A*2)	65	0.6970	122	0.7068
Food (*A*3)	299	0.1924	511	0.1563
Clothing (*A*4)	13	0.0505	17	0.0507
Residence (*A*5)	212	0.2007	1504	0.4103
Household equipment supplies (*A*6)	85	0.0594	360	0.1059
Communications (*A*7)	89	0.0875	202	0.1059
Culture, education and entertainment (*A*8)	103	0.0268	153	0.0181
Healthcare (*A*9)	56	0.0616	185	0.0836

**Table 6:** Income elasticity of regional residents.

Project		*A*2	*A*3	*A*4	*A*5	*A*6	*A*7	*A*8	*A*9
Sample period	1	1.0328	0.5917	1.1762	1.8856	2.1082	1.9565	0.3722	1.5136
	2	1.0694	0.6079	1.1938	1.9048	2.3695	1.8462	0.4073	1.6554
	3	1.0577	0.6431	1.0984	1.8120	2.0547	1.1571	0.4323	1.4378
	4	1.0197	0.6887	1.0293	1.4740	1.8448	1.2911	0.4392	1.4247
	5	1.0033	0.7287	1.0238	1.2550	1.6824	1.2530	0.4870	1.3716
	6	1.0199	0.7349	1.0711	1.2537	1.5563	1.3393	0.5578	1.2761
	7	0.9889	0.7581	1.0741	1.1013	1.3997	1.3568	0.5504	1.2186
	8	1.0457	0.7752	1.0647	1.4492	1.2918	1.2054	0.5922	1.1815

Forecast period	9	1.0277	0.6615	1.0552	2.2983	2.1000	1.4209	0.4291	1.5193
	10	1.0248	0.6863	1.0476	2.0691	1.9174	1.3657	0.4570	1.4479
	11	1.0220	0.7118	1.0437	1.8613	1.7476	1.3140	0.4886	1.3824
	12	1.0193	0.7381	1.0375	1.6770	1.5962	1.2634	0.5211	1.3180
	13	1.0163	0.7655	1.0316	1.5100	1.4572	1.2159	0.5562	1.2570

## Data Availability

The data used to support the findings of this study are available from the author upon request.
